# Denial and Misconceptions about Tropical Deforestation

**DOI:** 10.1093/biosci/biaf210

**Published:** 2026-01-23

**Authors:** Colin A Chapman, Carlos A Peres

**Affiliations:** Biology Department, Vancouver Island University, Nanaimo, British Columbia, V9R 5S5, Canada; Shaanxi Key Laboratory for Animal Conservation, Northwest University, Xi’an, 710069, China; School of Life Sciences, University of KwaZulu-Natal, Pietermaritzburg, 3209, South Africa; School of Environmental Sciences, University of East Anglia, Norwich, NR4 7TJ, UK; Instituto Juruá, Manaus, 69057-060, Brazil

Deforestation destroys some of the Earth’s most biodiverse regions. Despite their importance, forest destruction increased in 2023 beyond levels seen when 140 countries pledged to halt deforestation by 2030 (Forest Declaration Assessment 2025). In fact, 6.73 million hectares of primary forest were cleared globally in 2024, with most tropical regions failing to meet their targets (Forest Declaration Assessment 2025).

Unfortunately, although deforestation statistics often make headlines, the situation is surrounded by denial and misconceptions. This coincides with a rise in organized denial about both the biodiversity and climate crises (Lees et al. 2020) and an increase in misinformation about science in general (National Academies of Sciences Engineering and Medicine 2024). This is particularly well documented for climate change denial by the far right in the United States, Europe, and Brazil (Diele-Viegas et al. 2021, Forchtner 2019). Denial and misconceptions about deforestation take four forms (figure 1): outright denial, misconceptions about how current processes will shape future deforestation, misconceptions that reforestation can provide a quick fix, and denying the true environmental impact of deforestation.

**Figure 1.**
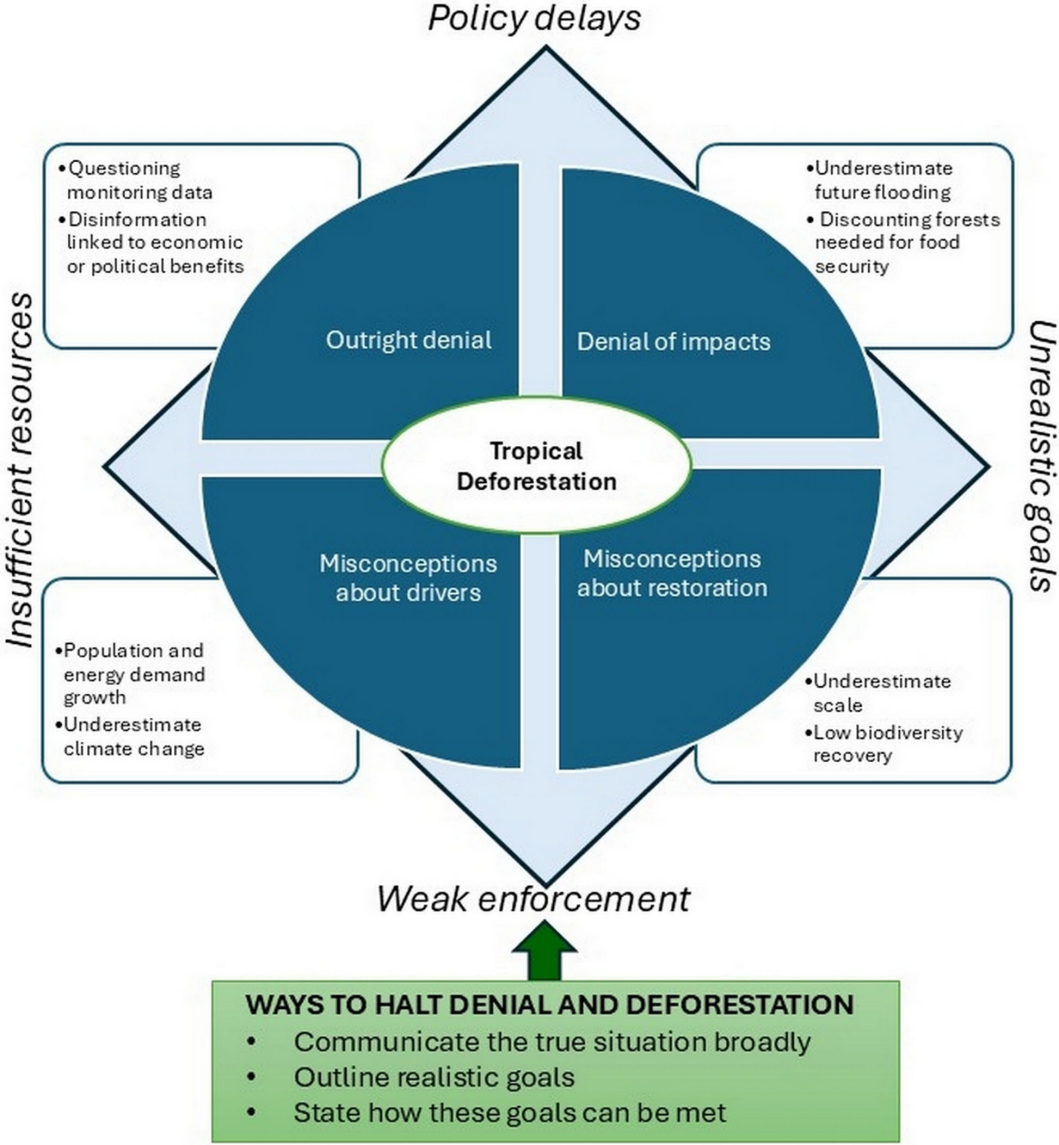
Four aspects of denial or misconceptions that facilitate continued tropical deforestation and some of their important components. These are ringed by factors that continue to allow deforestation unabated. Ways with which denial and therefore deforestation could be halted are shown in the box at the bottom.

## Outright denial

One of the clearest examples of outright denial concerns the data release from the National Space Research Institute (INPE) of Brazil showing that 1000 square kilometers of the rainforest had been cleared in the first 15 days of July 2019 and the then far-right president Jair Bolsonaro accusing them of exaggeration (BBC 2019). This occurred even though several scientific institutions, including the Brazilian Academy of Sciences, defended the INPE and the accuracy of its data.

Outright denial is often linked to those seeking economic or political benefits at the expense of forests. A recent report by the National Academies of Sciences, Engineering, and Medicine (2024) highlights the sharp rise in denial and misinformation attributed partly to poorly regulated online platforms and cuts in news outlets. Considering this report and that climate change and biodiversity denial are rising, we can expect to see more instances of outright denial of deforestation.

## Denial of the true environmental impact of deforestation

Many people do not grasp the full environmental consequences of deforestation because its effects take years to become apparent. For example, deforestation can lead to flooding long after trees are cut down, incurring major economic cost and human suffering. In 2024, exceptionally high rainfall in 27 tropical African countries, many of which have been extensively deforested, displaced 4 million people and caused 2500 fatalities (Africa Center for Strategic Studies 2024). Deforestation also contributes to gradual changes in the Earth’s climate and in 2024 deforestation and natural and anthropogenic fires were projected to have released 4.2 billion tons of carbon dioxide into the atmosphere (Global Carbon Budget 2024). Deforestation significantly contributes to biodiversity loss and regional extinctions, likely more than is acknowledged, as scientists are very conservative in declaring a species extinct (Lees et al. 2020). It also reduces water cycling and aquifer recharge. Finally, forests play a crucial role in poverty alleviation and global food security. Therefore, deforestation has widespread negative impacts on societal well-being from local to global scales, which often emerge years after forest loss.

## Misconceptions about how current processes will shape future deforestation

A common economic and political misconception is that deforestation declines as countries develop and become wealthier (Crespo et al. 2017). This belief is based on trends seen in North America and Europe and involves dubious economic thinking that suggests that as nations develop, environmental degradation initially increases but is reversed as per capita wealth and technology eventually ensure better environmental practices (Lees et al. 2020). However, these trends occur at the local level and likely are not applicable globally.

European countries and the United States are outsourcing the forest products they use, with direct consequences to deforestation in biodiverse nations in the Global South (Wiebe and Wilcove 2025). Globalization has increasingly allowed countries to externalize their environmental costs of land use. For example, since 1961, crops grown for export have expanded at twice the rate of crops grown for domestic consumption (Wiebe and Wilcove 2025). Agricultural expansion often come at the expense of forest loss in biodiverse tropical countries. This has resulted in most of global forest loss occurring in the tropics (Keenan et al. 2015). Furthermore, global wood production is at record levels, at about 4 billion cubic meters per year, around half of which is used for fuel primarily in the Global South (FAO 2024).

People often have a short-term perception of deforestation, ignoring future conditions. For example, the world’s population is projected to increase from the current 8.2 billion to 9.7 billion by 2050 with more than half of this growth occurring in Africa (United Nations 2024). Feeding and providing cooking fuel for the expanding population will lead to further deforestation. Estimates suggest that food production worldwide—usually made possible by hinterland cropland expansion—needs to increase by 1.1% each year (Bahar et al. 2020). In addition, per capita increases in purchasing power in many countries are translating into diets at higher trophic levels, thereby inflating the average agricultural land footprint. In Africa, where the greatest population growth will occur, fuelwood supplies over 80% of domestic energy needs, and, given the rural prevalence of the population, this is unlikely to change rapidly (Mayaux et al. 2013). Furthermore, trends in deforestation often overlook the impacts of climate change. One estimate suggests that 75% of all tropical forests remaining in 2000 will experience temperatures higher than those presently supporting closed-canopy forests by 2100 (Wright et al. 2009). This will increase pressure on any remaining forests to provide goods and services.

## The misconception that reforestation will provide a quick fix

Technological fixes are often advocated as silver bullets to solve complex environmental problems. In the context of deforestation, some dismiss its significance by asserting that restoration can remedy the situation. This is at the very least misleading. Restoration efforts are motivated by a diversity of worthy goals, including conserving biodiversity, sequestering carbon, improving water supply, and sustaining human likelihoods. However, they are insufficient to be considered a fix given the vast areas of deforested lands, the current rate of deforestation, and the costs and effectiveness of successful restoration efforts. For example, 6.37 million hectares of forest were lost in 2023 (Forest Declaration Assessment 2025); therefore, to merely maintain existing levels of forest cover, an area the size of Myanmar or Afghanistan would need to be reforested each year.

To express the scale of the needed effort in another way, consider existing government pledges. There are an estimated 3.4 billion hectares of deforested and degraded land globally (Forest Declaration Assessment 2025). Restoring only 30% of these ecosystems by 2030, as recommended by the Kunming–Montreal Global Biodiversity Framework, means restoring at least 1 billion hectares—an area equivalent to the size of Canada.

Most importantly, asserting that reforestation will provide a quick fix overlooks many complexities that make long-term reforestation gains uncertain. For example, once forests are degraded, they are typically occupied by local communities. As a result, groups engaging in tree-planting efforts frequently work with those communities, but these relations can deteriorate. In Uganda, a carbon-offset project involving local community members planting trees on their own land has resulted in adverse opportunity costs for local livelihoods, including families not having sufficient remaining farmland to grow food crops (Kamukama and Kamukama 2022). Such discontent can undermine restoration efforts. For example, in Brazil, hostilities arose because reforestation efforts conflicted with competing land interests and a forest restoration project in the Rio Preto–Jacundá Extractive Reserve was destroyed by fires set by land-grabbers (Prizibisczki 2023).

One should keep in mind that many reforestation efforts involve fast-growing tree plantations often with a few nonnative species, are in areas with inadequate seed banks, on community-owned land, or far removed from undisturbed forest. Therefore, their ability to foster biodiversity recovery is very low. Furthermore, many restoration efforts that are focused on carbon sequestration are misguided, because they do not consider the type of ecosystem in which restoration is implemented (e.g., grasslands; Pereira et al. 2024).

A general impression can be made by evaluation progress in meeting international restoration pledges. In 2022, 200 nations signed an ambitious agreement to halt and reverse biodiversity loss, but none were on track by the COP16 in February 2025. In fact, only 36 countries had submitted the required national biodiversity strategies and action plans, but none had plans to reach commitments for target 2 (the 30% restoration target) and target 3 (the 30% protection target). Only nine nations had committed to restoring a specific percentage of land and sea, and only six had committed to the agreed-on restoration level of 30% (Bell-James and Watson 2025). This suggests that the likelihood of a significant proportion of the signatory countries creating and financing plans by 2030 is, at best, slim.

These observations should not discourage reforestation efforts, because restoration has great potential to benefit specific regions, many endangered species, watersheds, carbon storage, and support local communities. However, it is important to keep in mind that restoring tropical forests, regardless of how much money is thrown at it, results in a poor outcome in terms of biodiversity and carbon value compared with simply retaining old-growth forests.

## Conclusions

To design effective conservation strategies to reduce deforestation, scientists must combat denial and address misconceptions. It is not enough to conduct rigorous science and publish in scientific journals; information must be communicated broadly to the public and policymakers. This will require the involvement of journalists, influencers, and bridgers. Only through effective communication can the message of those who actively or passively deny deforestation and its consequences be countered and misconceptions corrected. It is important to not only detail the extent of losses but also communicate the multifaceted ramifications of this loss. This will be most effective when scientists demonstrate connections that resonate with the target audience. This can be done, for example, by demonstrating difficulties local communities will face regarding food security and finding cooking fuel if uncontrolled deforestation occurs; this requires field work and community connections.

Some misconceptions arise because scientists have a strong desire to make positive impacts and propose solutions without grasping the scale and complexities of the problem. For instance, advocating that restoration can be a quick fix for deforestation overlooks the massive annual losses and reforestation costs and the typically low biodiversity value of reforested sites compared with old-growth forests. A clear example of not considering the complexity of the situation is when reforestation efforts do not take local community needs into considerations—not just their current needs but how their needs will change over the decades that the reforestation effort must last.

Scientists are faced with the dilemma of seeking to encourage positive conservation action but having to do so in a realistic fashion that will not promote misconceptions. On one hand, it is widely recognized that presenting a positive, optimistic message (e.g., "we are all in this together, and we can make positive change") is more likely to lead to action than a doom-and-gloom perspective. On the other hand, it is important to relay a realistic portray of the situation (e.g., "with our current resources, we can reach this specific goal"). The way out of this dilemma is to set realistic objectives and to optimistically state how reaching those goals will be advantageous. Scientists must inspire action while remaining grounded in solid science directed at realistic goals. They must strive to be both hopeful and truthful, crafting messages that acknowledge the daunting challenges but that highlight achievable steps forward.
